# Das Bertolotti-Syndrom: eine unterdiagnostizierte Ursache für spezifischen Rückenschmerz

**DOI:** 10.1007/s00132-025-04656-1

**Published:** 2025-04-29

**Authors:** Anna-Lena Hauser, Alexander Von Glinski, Javier Fernando Noriega Urena, Tobias Lange, Samira Murad, Guido Lewik, Tobias Schulte

**Affiliations:** https://ror.org/046vare28 grid.416438.c St. Josef-Hospital Universitätsklinikum der Ruhr-Universität Bochum, Gudrunstraße 56, 44791 Bochum, Deutschland

**Keywords:** Konservative Behandlung, Verzögerte Diagnose, Wirbelsäulenerkrankung, Spinale Fusion, Radikulopathie, Conservative treatment, Delayed diagnosis, Spinal diseases, Spinal fusion, Radiculopathy

## Abstract

**Hintergrund:**

Lumbosakrale Übergangsanomalien (LSTV; Bertolotti-Syndrom) sind mit einer Prävalenz von 16–35 % häufig und können eine Ursache spezifischen Rückenschmerzes sein. Die unzureichende Bekanntheit dieser Anomalie führt oft zu einer verzögerten Diagnosestellung und Therapie.

**Ziel der Arbeit:**

Diese Arbeit erläutert die anatomischen und biomechanischen Grundlagen des Bertolotti-Syndroms und diskutiert Diagnostik- und Therapiemöglichkeiten.

**Material und Methoden:**

Diese systematische Übersichtsarbeit wurde gemäß den PRISMA-Richtlinien (Preferred Reporting Items for Systematic Reviews and Meta-Analyses) durchgeführt. Die SCOPE-Kriterien (Structuring Comparative Outcome Reporting in Epidemiology) wurden berücksichtigt, um eine umfassende und transparente Berichterstattung zu gewährleisten. Eine systematische Recherche wurde in PubMed mit den Suchbegriffen „Bertolotti“ und „Bertolotti syndrome“ und „lumbosacral transitional vertebrae“ durch eine Untersucherin durchgeführt und 112 Studien eingeschlossen. Artikel, die nicht in englischer oder deutscher Sprache verfügbar waren, wurden ausgeschlossen.

**Ergebnisse:**

Patienten mit einem symptomatischen Bertolotti-Syndrom leiden häufig an chronischen lumbalen Rückenschmerzen, ggf. mit Radikulopathien. Bildgebende Verfahren, insbesondere Röntgen, MRT und CT, spielen eine Schlüsselrolle bei der Diagnose. Konservative Therapien zeigen nur begrenzten Erfolg, v. a. in Fällen, in denen die Fehlbildung nicht in das Therapiekonzept integriert wird. Operative Alternativen umfassen die operative Entfernung des Neoarthrosspaltes, eine Fusion des betroffenen Segmentes und im Einzelfall auch Neuromodulationstechniken.

**Diskussion:**

Die Wahl der Behandlung hängt von der Beschwerdekonstellation des Patienten, der individuellen Anatomie und dem Vorhandensein symptomatischer degenerativer Veränderungen von Neoarthros, Facettengelenken, Bandscheiben, Iliosakralgelenk und Neuroforamen ab. Während konservative Maßnahmen initial empfohlen werden, zeigt die Processektomie bei sorgfältiger Auswahl der Patienten vielversprechende Ergebnisse. Fusionsoperationen und Neuromodulationstechniken sind Alternativen. Die vorhandene Evidenz ist spärlich. Weitere Studien sind notwendig, um die Wirksamkeit der einzelnen Therapieoptionen für bestimmte Subgruppen besser bewerten zu können.

## Abstract (en)

**Background:**

Lumbosacral transitional vertebrae (LSTV) are common, with a prevalence of 15–35%, and can be a cause of specific low back pain. The insufficient awareness of this anomaly often leads to delayed diagnosis and treatment.

**Objective:**

This study explains the anatomical and biomechanical basis of Bertolotti syndrome and discusses diagnostic and therapeutic options.

**Materials and methods:**

This systematic review was conducted in accordance with the PRISMA guidelines (Preferred Reporting Items for Systematic Reviews and Meta-Analyses). The SCOPE criteria (Structuring Comparative Outcome Reporting in Epidemiology) were considered to ensure comprehensive and transparent reporting. A systematic search was performed in PubMed using the search terms ‘Bertolotti’, ‘Bertolotti syndrome’, and ‘Lumbosacral transitional vertebrae’ by a single investigator, resulting in the inclusion of 112 studies. Articles that were not available in English or German were excluded.

**Results:**

Patients with symptomatic Bertolotti syndrome often suffer from chronic low back pain and radiculopathies. Imaging techniques, particularly X‑rays and MRI, play a key role in diagnosis. Conservative treatments show limited success. Surgical resection of the transverse process (processectomy), especially after positive test infiltrations, leads to significant pain relief, particularly in younger patients without degenerative changes.

**Discussion:**

Treatment choice depends on individual anatomy and the presence of degenerative changes. While conservative measures are initially recommended, processectomy shows promising results in carefully selected patients. Fusion surgeries should only be considered in cases of instability. Further studies are needed to confirm the effectiveness of invasive procedures.

Das Bertolotti-Syndrom ist eine häufige angeborene lumbosakrale Übergangsanomalie (LSTV) mit einer Prävalenz von 15,8–35,6 % und kann Schmerzen im Lumbosakralbereich sowie Radikulopathien verursachen. Oft dauert die Diagnose lange, da das Syndrom wenig bekannt ist und die Rolle des LSTV als Schmerzursache lange umstritten war [1].

## Anatomie und Pathogenese

Lumbosakrale Übergangsanomalien sind nicht per se pathologisch und häufig asymptomatische Zufallsbefunde. Ursächlich für ihre Entstehung ist eine Störung der für die Segmentation verantwortlichen chemotaktischen Signale, die in der 6. Schwangerschaftswoche die Fusion und Chondrifikation der bis dahin angelegten vertebralen Strukturen koordinieren [2, 3]. In unterschiedlichen Ausprägungsformen resultiert eine Sakralisation des 5. Lendenwirbelkörpers oder Lumbalisation des 1. Sakralwirbelkörpers [4].

Es zeigt sich eine laterale und rostrale Vergrößerung des Processus transversus des LWK 5 bei proximaler reichenden Alae sacrales [5]. Ein Kontakt der Strukturen im Sinne eines Neogelenkes resultiert, der sich auch in histologischen Gewebeveränderungen analog einer Knorpeldegeneration widerspiegelt. Klinisch kann dies als *lumbosakraler Rückenschmerz* auffällig werden, in der Regel nach gluteal ausstrahlend [4].

In Einzelfällen kann es durch die „Arthrose“-Bildung am Neogelenkspalt medial zu Osteophytenbildung kommen, die ihrerseits nach medial ins Neuroforamen L5/S1 ragen und dort eine *L5-Radikulopathie* auslösen können [6]. O. g. Inflammation im Rahmen einer Arthritis des Neogelenkes kann die Wurzel außerdem chemotaktisch reizen [4]. Eine extraforaminale L5-Reizung durch LSTV wurde von Wiltse als „far out syndrome“ erstbeschrieben [7, 8]. Im Falle einer Lumbalisation des SWK 1 kann es statt zu einer L5- zu einer *S1-Symptomatik* kommen, wenn nämlich das Neogelenk in Höhe S1/S2 vorliegt [1].

Das Iliosakralgelenk (ISG) wird durch den Bertolotti-Spalt (Neogelenkfuge) kraniokaudal zweigeteilt. Durch diese Asymmetrie ist eine funktionelle Störung des gesamten ISG-Komplexes anzunehmen und als Ursache eines *lokalen ISG-Schmerzes* wahrscheinlich [1, 4].

Das L5/S1-Segment ist durch den LSTV insb. in Hinblick auf die Lateralflexion und axiale Rotation rigider bis völlig immobil. Dies hat zwei klinisch relevante Konsequenzen. Zum einen resultiert eine Entlastung der Bandscheibe L5/S1, weswegen diese auch im fortgeschrittenen Alter häufig sehr wenige bis gar keine Degenerationsphänomene aufzeigt, was häufig in keiner Weise zur Degenerationssituation der restlichen LWS passt. Zum anderen resultiert aus der Fehlbildung, dass das erste frei bewegliche Segment statt L5/S1 in diesen Fällen das Segment L4/5 ist. Die klassischen Degenerationsprozesse (Osteochondrose, Bandscheibenvorfall, degenerative Instabilität etc.) entstehen v. a. in diesem Segment L4/5 aufgrund der konsekutiven relativen Überlastung und Hypermobilität [9]. Auch ist das sonst für die lumbosakrale Stabilität bedeutsame Lig. iliolumbale bei Bertolotti-Patienten eher schwach ausgebildet. Es kommt häufiger und in jüngerem Lebensalter zu Bandscheibenvorfällen, Foramenstenosen, Facettengelenksarthrose sowie degenerativen Lumbalskoliosen [10]. Hieraus können sekundär Radikulopathien sowie lokaler Rückenschmerz der kranialeren Segmente entstehen [6, 11].

Im Falle einer unilateralen Fusion ist zumindest eine relevante Asymmetrie der Belastung lumbosakral als gesichert anzunehmen. Fehlbelastungen und ungewohnte Degenerationsprozesse auf der kontralateralen Seite sind realistisch, sowohl im Bereich der Facettengelenke als auch im Bereich der ISG. Auch die Funktion des ipsilateralen Facettengelenkes ist durch ein Neogelenk im Vergleich zum „normalen Zustand“ verändert, was jeweils zu lokalen Schmerzen führen kann.

Eine *myogelotische Schmerzkomponente* durch kompensatorische Myogelosen des M. quadratus lumborum und M. iliopsoas sowie auch der tiefen und oberflächlichen posterioren Rückenmuskulatur kann ergänzend hinzutreten [9].

Bertolotti-Patienten sind häufig jünger [9] bis hin zum Kindesalter [12] und haben eine signifikant höhere Schmerzbelastung als in der altersgematchten Vergleichsgruppe [13]. Ebenso leiden sie häufiger an psychiatrischen Erkrankungen [13] ggf. in Zusammenhang mit der psychosozialen Belastung durch die zumeist verzögerte Diagnosestellung [14].

Die LSTV werden trotz ihrer hohen Prävalenz in ihrer klinischen Bedeutung noch nicht adäquat im Alltag gewürdigt und beachtet. Die Fehlbildung erklärt sicherlich nur bei einem Teil der Träger deren klinische Beschwerden. Ein vollständiges Ignorieren der Fehlbildung verkennt andererseits die Fälle, bei denen sie eindeutig die Ursache der Schmerzen sind, was häufig zu langen Leidenswegen führt. Ein realistisches Beachten des Bertolotti-Syndroms sowohl im diagnostischen als auch im therapeutischen Kontext sollte bei relativ schwacher Evidenzlage empfohlen werden.

## Einteilung und Klassifikation

**Abb. 1:**
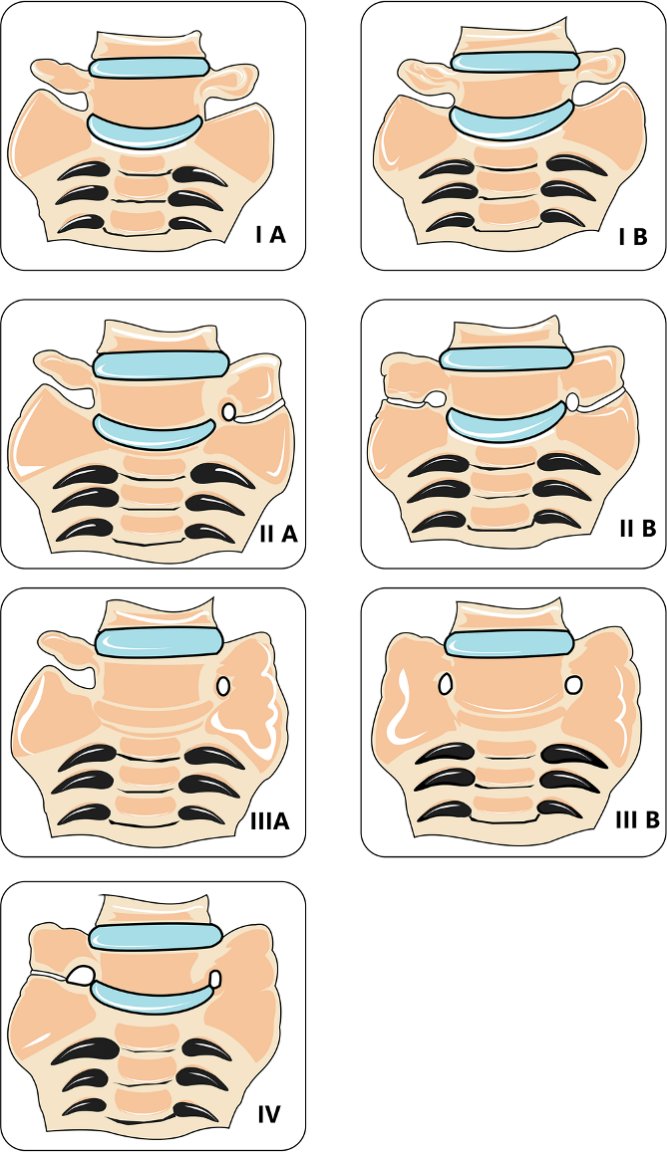
Formen des Bertolotti-Syndroms nach Castellvi

Nach Castellvi et al. wird das Bertolotti-Syndrom in 4 Subtypen unterschieden (Abb. 1). Typ 1 weist einen vergrößerten, triangulären Processus transversus (kraniokaudale Ausdehnung des Querfortsatzes ≥19 mm) auf. Typ 2 zeigt ein Neogelenk zwischen dem Processus transversus des L5 und der Ala ossis sacri. Zusätzlich wird zwischen Typ A (unilateraler Befund) und Typ B (bilateraler Befund) unterschieden. Bei Typ 3 liegt eine Verknöcherung vor, d. h. es besteht kein Neogelenkspalt mehr, stattdessen findet sich eine solide Fusionsmasse. Ergänzend gibt es bei Typ 4 noch eine Mischung der Befunde aus Typ 2 einerseits und Typ 3 andererseits [15].

## Diagnostik

Für die Anamnese ist oft eine lange Leidensgeschichte typisch.

Klinisch steht ein lokaler lumbosakraler Schmerz mit Ausstrahlung nach gluteal im Vordergrund, der häufig unter mechanischer Belastung zunimmt und in Ruhe bei Entlastung weniger ausgeprägt ist. Wie oben beschrieben, kann eine L5- oder S1-Radikulopathie hinzutreten.

Einen typischen Test für das Neogelenk, d. h. den Bertolotti-Spalt, gibt es nicht. Die klinische Untersuchung sollte die Lendenwirbelsäule, die ISG (u. a. lokaler Druckschmerz, Patrick-Test = FABER-Test = Flexion, Abduktion und Außenrotation und Gaenslen-Test), die Hüftgelenke (u. a. Thomas-Test, Impingement-Test [FADIR-Test = Flexion, Adduktion und Innenrotation] und Trendelenburg-Test) und die lumbalen Nervenwurzeln (v. a. L5 und S1) abklären. Auch Tests wie der Multifidus-Lift-Test und der Prone-Instability-Test sind sinnvoll zur Eingrenzung der Beschwerden.

Paresen sind eher untypisch, es sei denn sie basieren z. B. auf einem akuten Bandscheibenvorfall im ersten freien Bewegungssegment.

Bei persistierenden Beschwerden sollte die Bildgebung initial aus einer Röntgenaufnahme der LWS im Stand in 2 Ebenen bestehen. Sie weist eine bis zu 84 %ige Sensitivität in der Detektion von LSTV und eine bis zu 58 %ige Sensitivität in der korrekten Klassifikation auf [16]. Als röntgenologische Spezialaufnahme kann bei unklaren Befunden eine Ferguson-Aufnahme (a. p. in 30° Lordose) ergänzt werden [6, 9].

**Abb. 2:**
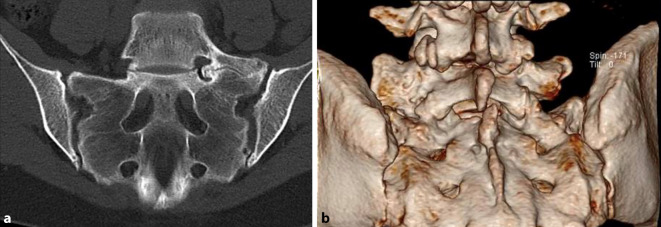
CT-Darstellung (**a**) einer lumbosakralen Übergangsanomalie Typ Castellvi IIA mit 3‑D-Rekonstruktion (**b**)

Eine MRT der LWS und des Sakrums dient dem Ausschluss lumbaler Differenzialdiagnosen, allen voran des ersten freien Bewegungssegmentes sowie dem Nachweis aktivierter Neogelenkbereiche (z. B. in STIR-Sequenzen). Eine CT des LSTV dient der optimalen dreidimensionalen Darstellung der knöchernen Anomalie inkl. möglicher Osteophyten im Neuroforamen (Abb. 2).

**Abb. 3:**
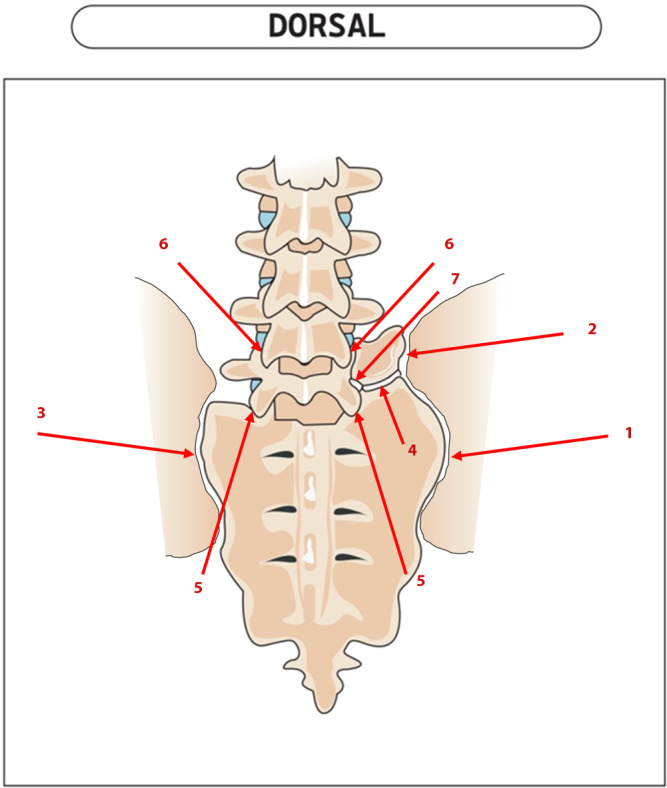
Infiltrationsmöglichkeiten. *1* Iliosakralgelenk (ISG) kaudal des Neoarthros, *2* ISG kranial des Neoarthros, *3* ISG der Gegenseite, *4* horizontaler Neoarthros, *5* Facettengelenk L5/S1 ipsi- und kontralateral, *6* Facettengelenke L4/5 und *7* Nervenwurzel L5 intraforaminal L5/S1

Bei radiologischem LSTV-Nachweis mit passender Klinik sind diagnostische Infiltrationen der potenziellen Schmerzgeneratoren hilfreich, wenngleich es keine publizierte Evidenz zu ihrer Wertigkeit gibt (Abb. 3). Die Besserungsrate jeder einzelnen Infiltration ist sicherlich nicht absolutistisch als klarer *Be*weis für oder gegen eine Struktur als Schmerzursache zu werten, das Konzept der Infiltrationsabklärung kann aber dennoch wertvolle *Hin*weise liefern, um das weitere Vorgehen beim individuellen Patienten zu planen.

Als Besonderheit sei erwähnt, dass nicht in jedem Fall eine Nadelplatzierung *im* Bertolotti-Spalt möglich ist, selbst wenn in der CT ein Spalt sichtbar ist, da nicht selten dorsal des Spaltes Knochenbrücken bzw. Osteophyten den direkten Weg in den Spalt erschweren.

## Therapeutische Möglichkeiten

### Konservative Therapie

**Abb. 4:**
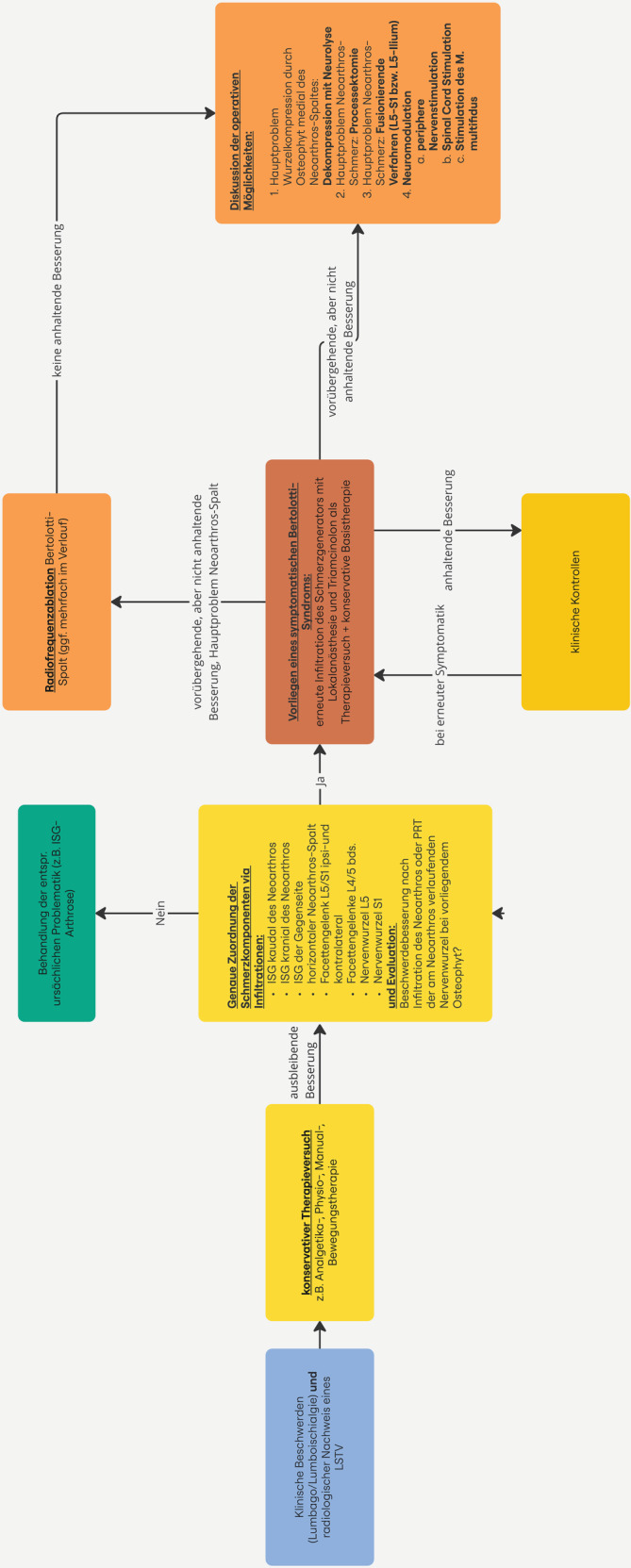
Diagnostischer und therapeutischer Algorithmus; *ISG* Iliosakralgelenk, *LSTV* lumbosakrale Übergangsanomalie

Alle Aspekte der konservativen Wirbelsäulenorthopädie (Analgetika, Physiotherapie, physikalische Therapie und Manualtherapie, Osteopathie, Bewegungs- und Sporttherapie, Akupunktur sowie Stoßwellentherapie der paravertebralen Muskulatur [1]) können grundsätzlich zum Einsatz kommen (Abb. 4). Evidenz für zielführende nichtinvasive Schmerztherapie ist rar und geht nicht über Fallberichte hinaus.

Zudem sind röntgenkontrollierte Infiltrationen der im Abschnitt Diagnostik genannten Strukturen ein diagnostisch wichtiger Pfeiler, die dann bei Erfolg auch therapeutisch zum Einsatz kommen. Es gibt keinen Konsens darüber, welche Strukturen in welcher Reihenfolge infiltriert werden und ob Steroide zusätzlich zum Lokalanästhetikum verwendet werden sollten. Die Rate an schmerzfreien oder -gebesserten Patienten sowie der Wirkungszeitraum variieren stark, von wenigen Stunden bis zu Jahren [17]. Die meisten Fallberichte zeigen, dass nur wenige Patienten eine dauerhafte Schmerzreduktion durch rein konservative Therapien inkl. Infiltrationen erreichen.

Es bestehen große Unterschiede im weiteren Vorgehen. Die Spannbreite der Interpretation eines positiven Infiltrationsergebnisses reicht vom Absehen von weiteren Behandlungen, da die Schmerzfreiheit als Behandlungsziel interpretiert wird und dieses zumindest vorerst erreicht ist, bis hin zur Stellung der Operations- bzw. Denervierungsindikation, da die Infiltrationen als diagnostischer Pfeiler und nicht als therapeutisches Mittel verstanden werden.

### Denervierung

Wird der Bertolotti-Spalt selbst oder ein Teil des ISG oder ein Facettengelenk durch eine positive diagnostische Testinfiltration als (Teil‑)Ursache des Rückenschmerzes eruiert, kann eine lokale Denervierung dieser Struktur sinnvoll sein. Zielstruktur sind Äste der Rami posteriores der entsprechenden Spinalnerven. Die wenigen vorliegenden Studien zu diesem Thema zeigen in sich uneinheitliche Ergebnisse. Positive Probeinfiltrationen werden nicht in allen Studien einheitlich vorausgesetzt.

Zudem wird mit unterschiedlichen Methoden (klassische Thermodenervation, gepulste/gekühlte/bipolare Radiofrequenzablation) gearbeitet. Selbst innerhalb der gleichen Methoden variieren Parameter wie die Dauer der Intervention, die Zieltemperatur und die lokalen Abstände der Denervationssonden bei bipolaren Techniken stark. Schlussendlich kann man die publizierten Protokolle mit jeweils geringen Fallzahlen also nur bedingt vergleichen und die naturgemäß damit stark differierende Schmerzlinderung nur eingeschränkt bewerten.

Burnham et al. veröffentlichten 2010 den ersten Fallbericht einer in einer Schmerzreduktion resultierenden Denervierung [18]. Nach erfolgreicher Probeinfiltration wurde zirkulär um das Neogelenk herum bipolar denerviert. 4 Tage nach der Intervention ebbte der Interventionsschmerz ab und eine 16 Monate anhaltende 100 %ige Schmerzreduktion wurde erzielt [18].

Jain et al. ablatierten bei 20 Patienten sowohl für das ISG, die Facettengelenke und Rami communicantes für eine Dauer von 90 s nach positiver Probeinfiltration (Schmerzbesserung >80 %) und boten Patienten mit neurogener Claudicatio oder neurogenem Beinschmerz bei Bandscheibenvorfällen im kranialen Segment eine gepulste Radiofrequenzablation der Nervenwurzel bei 42 °C für 4 min an. Bei allen Patienten konnte eine Schmerzbesserung für mindestens 6 Monate berichtet werden. Das Neogelenk selbst wurde in dieser Studie nicht radiofrequenzablatiert [19].

In unserer Klinik setzen wir mindestens zwei positive Probeinfiltrationen der o. g. potenziellen Schmerzgeneratoren insbesondere auch des Neogelenkes für eine Denervierung voraus.

### Operative Therapiemöglichkeiten

Die chirurgischen Therapiemöglichkeiten sind vielfältig und richten sich nach der herausgearbeiteten Schmerzursache.

Steht der lumbosakrale Rückenschmerz im Vordergrund, kann eine operative Resektion des Neogelenkes (Processektomie) erwogen werden. Diese kann minimalistisch durch Fräsen einer Schneise im Spaltbereich erfolgen oder maximal eine vollständige Entfernung des Querfortsatzes L5 bedeuten.

Steht eine Radikulopathie der Nervenwurzel, die im Foramen auf Höhe des Neogelenkspaltes durch einen Osteophyten irritiert ist, im Vordergrund, so kann eine Foraminotomie sinnvoll sein.

Gegebenenfalls ist eine Kombination dieser Techniken notwendig.

Als Ultima Ratio besteht die Möglichkeit, das betroffene Segment (L5/S1) mittels posteriorer Instrumentationsspondylodese zu fusionieren.

Lässt sich diagnostisch das ISG als relevante Rückenschmerzursache mit herausarbeiten, kann im Einzelfall auch eine Fusion des ISG denkbar sein, ggf. auch in Kombination mit der Fusion L5/S1.

Wenn das erste bewegliche Segment L4/5 degeneriert ist und mit hoher Wahrscheinlichkeit am Schmerz beteiligt ist, sollte dies Segment nach den üblichen Therapiealgorithmen unabhängig vom Bertolotti-Syndrom behandelt werden.

Eine retrospektive Studie aus den Jahren 1974–1987 verglich erstmals die Outcomes von je 16 konservativ und 16 operativ (8 Processektomien, 8 Fusionen) behandelten Patienten. Sie wird oft zitiert, um zu zeigen, dass operative Therapien konservativen nur leicht überlegen seien, weist aber erhebliche methodische Mängel auf. Die konservative Vergleichsgruppe wurde weder randomisiert noch nach definierten Kriterien gematcht noch wurde präoperativ per Schnittbildgebung ein LSTV nachgewiesen. Nur 6 der 16 operierten Patienten erhielten eine Probeinfiltration, (davon gaben 4 Patienten eine Schmerzbesserung an). Zudem waren heute gängige diagnostische und therapeutische Techniken (Operationsmikroskopie, Neuromonitoring, Navigation) nicht verfügbar. 5 der 8 Fusionspatienten und 5 der 8 Processektomiepatienten verbesserten ihre Rückenschmerzen. 7 der insgesamt 16 operierten Patienten wurden schmerzfrei bzgl. der Rückenschmerzen (keine Differenzierung nach Operationsverfahren). 11 von 13 Patienten mit Ischialgien hatten diese auch postoperativ, was aus unserer Sicht am ehesten auf die unzureichende Diagnostik zurückzuführen ist. 3 von 8 der fusionierten (2 Pseudarthrosen, 1 Bandscheibenvorfall) und 3 von 8 der processektomierten Patienten (1 Bandscheibenvorfall, 1 Re-Resektion, 1 anteriore Fusion) mussten revidiert werden [20].

Chang et al. konstatierten in einer aktuellen Metaanalyse hingegen, dass 118 der 138 (davon 130 Processektomien, 8 Fusionen) Patienten ein zufriedenstellendes Operationsergebnis aufwiesen, ohne dieses klar zu definieren [21]. Bei 18 Patienten führte die Processektomie und bei 2 Patienten die posteriore Fusion nicht zur gewünschten Schmerzlinderung. Inkonsequente Diagnostik stellte sich als häufiger Grund für chirurgische Misserfolge bei Processektomien heraus [21].

#### Processektomie

Eine Processektomie beinhaltet die Resektion des Neogelenkes. Diese Entfernung kann mit verschiedenen Techniken realisiert werden, sei es das Resezieren lediglich des Spaltes mit der Fräse, sei es das vollständige Entfernen des kranialen vergrößerten Querfortsatzes.

Wie weit in Richtung Foramen zu resezieren ist und ggf. eine echte Foraminotomie zu erfolgen hat mit Entfernung der Osteophyten, die die Wurzel komprimieren, ist abhängig von der klinischen Situation der Wurzel. Bei Fehlen einer Radikulopathie muss keine Foraminotomie erfolgen.

Auch kann diskutiert werden, wenn man den Neogelenkspalt mit der Fräse nach anterior entfernt, ob zum Schutz der Beckengefäße eine minimale Kortikalislamelle ventral bestehen bleiben kann.

Ein weiterer ungeklärter Aspekt betrifft die langfristigen Folgen der Teilresektion, z. B. der Technik der Schaffung einer Schneise mit der Fräse. Langfristig bildet sich trotz Nutzung von Knochenwachs zur Blutstillung in dieser Defektzone Narbengewebe, welches auch fusionieren kann. Ob dies Fusionsgewebe klinisch nachteilig in Erscheinung tritt, kann nur spekuliert werden. Es könnte sogar sinnvoll sein, Eigenknochen oder Knochenersatzmaterial hier im Bereich der Schneise einzubringen, um eine solide geplante Fusion zu induzieren.

Studienübergreifend wird initial zu Probeinfiltrationen des Neogelenkes (s. oben) geraten. Nur bei positivem Ansprechen auf jene ist ein gutes bis sehr gutes Operationsergebnis zu erwarten [4, 5]. Zudem sollten Patienten noch keine bzw. allenfalls sehr milde degenerative Veränderungen der angrenzenden Segmente aufweisen, da diese zuallermeist die Ursache für Schmerzrezidive darstellten [21–24]. Ju et al. verweisen auf die Relevanz insbesondere der Intaktheit der Bandscheibe L4/L5 [23].

Perioperative Komplikationen sind selten und umfassen drainagepflichtige Wundhämatome [24] und Wundheilungsstörungen [25]. In seltenen Fällen kann sich nach einer Processektomie eine solide Knochenbrücke zwischen dem Restquerfortsatz L5 und der Ala sacralis bilden und im ungünstigsten Fall erneut Wurzelkontakt verursachen [24]. Zudem sind in der Literatur L5-Radikulpathien nach Processektomie beschrieben [24, 25].

Die postoperativen Ergebnisse in der Literatur sind aufgrund variabler Diagnostik und Indikationsstellung nur begrenzt vergleichbar. McGrath et al. berichten von einer signifikanten Verbesserung der Lebensqualität nach Processektomie [26]. Jönsson et al. verzeichnen 64 % schmerzfreie und 18 % schmerzgelinderte Patienten (*n* = 11) [22], während Ju et al. 50 % Schmerzbesserung nach Processektomie und 72 % nach zusätzlicher Wurzeldekompression bei positiver Probeinfiltration beschreiben [23]. Li et al. berichten von 43 % dauerhafter Schmerzfreiheit und 29 % dauerhafter Schmerzlinderung (*n* = 7) insbesondere bei radikulärer Symptomatik [24]. Bei Castellvi II mit weitem Neogelenk wurden postoperativ vermehrt Instabilitäten berichtet [23]. Besonders bei Kindern wird eine anhaltende Schmerzbesserung ohne perioperative Komplikationen nach Processektomie beobachtet [12]. Dies bestätigt wiederum die Theorie, dass die Abwesenheit degenerativer Veränderungen ein guter Prädiktor für chirurgischen Erfolg ist. Postoperativ wird zu freier Mobilisierung bei mindestens 6‑wöchiger Sportkarenz und Ausbleiben von schwerem Tragen und Heben geraten [27].

#### Fusion

Wenn im Zusammenhang mit einem Bertolotti-Syndrom über „Fusion“ gesprochen wird, so ist zunächst zu klären, was für eine Fusion gemeint ist. Das direkte LSTV-Segment kann Ziel der Fusion sein, d. h. das Segment L5/S1.

In der Literatur sind die Ergebnisse fusionierender Verfahren der Processektomie unterlegen; bei korrekt indizierter Processektomie zeigen sich bessere postoperative Ergebnisse [28]. Golubovsky et al. wiesen nach, dass eine Fusion von L4–S1 bei LSTV spinale Torquemomente verstärkt und die Mobilität angrenzender Segmente erhöht. Wird nur das LSTV-Segment L5/S1 fusioniert, erhöht sich die Segmentmobilität in L3/L4 signifikant und tendenziell auch in L4/L5. Nur bei nachgewiesener deutlicher Degeneration oder schmerzprovozierender Instabilität von L5/S1 raten sie zur Fusion, da hierdurch insb. die in Lateralflexion einwirkenden Kräfte signifikant verringert werden konnten [11]. Deshalb sollte insbesondere bei jungen Patienten die Indikation zur Fusion äußerst zurückhaltend gestellt werden und als Ultima Ratio verstanden werden.

Mikula et al. konstatierten in einem überproportional alten und damit wahrscheinlich verstärkt degenerativen Veränderungen ausgesetzten Kollektiv (40±16 Jahre), dass Patienten mit Fusion langfristig häufiger schmerzgebessert waren als nach Processektomie (*n* = 27, 18 Processektomien, 9 Fusionen). Die Fusionsstrecke differierte hier jedoch stark (1 L3–S1, 2 L4–S1, 6 L5–S1), ebenso die Art der Fusion (3 ALIF, 1 TLIF, 5 posterolaterale Instrumentation), was die Vergleichbarkeit deutlich einschränkt [29]. In einem Fallbericht zeigte zudem eine Patientin, die jeweils v. a. bei Flexionsbewegungen der betroffenen Seite Beschwerden aufwies, eine gute Schmerzbesserung nach Fusion via PLIF und vorheriger positiver Probeinfiltration [30].

Im Falle einer Degeneration des kranialen Anschlusssegmentes (L4/5) sollte der Zustand dieses Anschlusssegmentes entscheiden, ob es als Ultima Ratio fusioniert werden könnte. Diese Entscheidung hat primär nichts mit dem darunter liegenden Bertolotti-Syndrom zu tun. Es ist aber zu bedenken, dass die im kaudalen Anschlussbereich jeder Fusion veränderten biomechanischen Verhältnisse sich durchaus auch negativ auf das Bertolotti-Segment auswirken können, was ggf. zur Empfehlung einer Fusion L4–S1 bei primär schmerzhaftem Segment L4/5 führen kann. Liegt eine isolierte Pathologie des kaudalen ISG auf der Seite der LSTV vor, kann als Ultima Ratio auch eine Fusion des betroffenen ISG diskutiert werden.

#### Neuromodulation

Als Alternative zur konservativen Therapie und zur reparativen operativen Therapie (Processektomie, Fusion) kann in Einzelfällen die Neuromodulation sinnvoll zum Einsatz kommen, sei es eine periphere Nervenfeldstimulation, ggf. eine Spinal-Cord-Stimulation oder auch die Stimulation der Mm. multifidi zur Stabilisierung der LWS.

Das operative und perioperative Risiko der Neuromodulationstechniken ist im Vergleich zu den beschriebenen Fusionstechniken relativ gering.

Für den Einsatz dieser Techniken beim Bertolotti-Syndrom findet sich keine harte Evidenz, im Einzelfall können sie allerdings zu deutlichen Verbesserungen der Schmerzsituation führen.

## Kritische Reflexion, Limitationen und alternative Erklärungen

Die Heterogenität der eingeschlossenen Studien erschwert direkte Vergleiche zwischen den Therapieansätzen. Variationen in Studiendesigns, Patientenpopulationen und Diagnosestandards können die Interpretation der Ergebnisse beeinflussen. Zudem beruhen fast alle Studien auf einer retrospektiven Datenanalyse mit kleinen Fallzahlen, was ihre Aussagekraft einschränkt. Die Effektivität insb. konservativer Therapien ist zudem unzureichend erforscht. Die meisten Studien fokussieren sich auf chirurgische Behandlungsoptionen, während die Langzeitergebnisse nichtoperativer Maßnahmen weitgehend unbekannt sind.

Alternative Erklärungen für die beobachteten Therapieerfolge sind zu berücksichtigen. Beispielsweise könnte eine Verbesserung der Rückenschmerzen nach einer Fusion mit der Ruhigstellung instabiler oder degenerativ veränderter Segmente zusammenhängen, anstatt einer spezifischen Wirkung auf die lumbosakrale Übergangsanomalie selbst.

Zukünftige Forschung sollte sich auf die prospektive Untersuchung verschiedener Therapieansätze konzentrieren, um evidenzbasierte Empfehlungen für die Behandlung des Bertolotti-Syndroms zu ermöglichen. Zudem sollten nicht nur Schmerzreduktion, sondern auch funktionelle Ergebnisse, die Prävention akzelerierter Degeneration und der Erhalt der Lebensqualität der Patienten in den Mittelpunkt zukünftiger Studien rücken.

## Fazit für die Praxis

- Das Bertolotti-Syndrom sollte als Ursache für spezifischen Rückenschmerz und ggf. Beinschmerz insb. bei jungen Patienten mit therapieresistentem Schmerz bedacht werden.
- Bildgebende Diagnostik ist sinnvoll.
- Stufenweise Testinfiltrationen sollten zur Identifikation des primären Schmerzgenerators folgen.
- Konservative Maßnahmen sollten ausgeschöpft werden, zeigen aber häufig keinen dauerhaften Erfolg.
- Bei positiven Testinfiltrationen kann eine Denervation sinnvoll sein.
- Die operative Entfernung des Neogelenkspaltes ist bei Abwesenheit relevanter konkurrierender degenerativer Ursachen der Nachbarsegmente zu erwägen.
- Eine Fusion des Segmentes der lumbosakralen Übergangsanomalie stellt eine Ultima-Ratio-Therapie dar.
- Techniken der Neuromodulation stellen im Einzelfall eine relativ risikoarme Alternative dar.

## Data Availability

An EndNote Library of the articles used for generating this review can be sent upon request. All articles are available via PubMed.

## Abkürzungsverzeichnis

ALIF: Anterior Lumbar Interbody Fusion
CT: Computertomographie
FADIR-Test: Flexion-Adduktion-Innenrotation-Test
FABER-Test: Flexion-Abduktion-Außenrotation-Test
ISG: Iliosakralgelenk
LSTV: Lumbosakrale Übergangswirbel (Lumbosacral Transitional Vertebrae)
LWK: Lendenwirbelkörper
MRT: Magnetresonanztomographie
OP: Operation
PLIF: Posterior Lumbar Interbody Fusion
STIR: Short Tau Inversion Recovery (MRT-Sequenz)
SWK: Sakralwirbelkörper
TLIF: Transforaminal Lumbar Interbody Fusion
a.p.: Anteroposterior (Vorder-Hinter-Ansicht beim Röntgen)
